# A Novel Health Information Technology Communication System to Increase Caregiver Activation in the Context of Hospital-Based Pediatric Hematopoietic Cell Transplantation: A Pilot Study

**DOI:** 10.2196/resprot.4918

**Published:** 2015-10-27

**Authors:** Molly Maher, David A Hanauer, Elizabeth Kaziunas, Mark S Ackerman, Holly Derry, Rachel Forringer, Kristen Miller, Dennis O'Reilly, Lawrence An, Muneesh Tewari, Sung Won Choi

**Affiliations:** ^1^ School of Information University of Michigan Ann Arbor, MI United States; ^2^ School of Public Health University of Michigan Ann Arbor, MI United States; ^3^ Department of Pediatrics University of Michigan Ann Arbor, MI United States; ^4^ Bioinformatics Core Comprehensive Cancer Center University of Michigan Ann Arbor, MI United States; ^5^ Department of Electrical Engineering and Computer Science University of Michigan Ann Arbor, MI United States; ^6^ Center for Health Communications Research University of Michigan Ann Arbor, MI United States; ^7^ Hematology/Oncology Division and Molecular Medicine and Genetics Department of Internal Medicine University of Michigan Ann Arbor, MI United States; ^8^ Department of Biomedical Engineering University of Michigan Ann Arbor, MI United States; ^9^ Biointerfaces Institute University of Michigan Ann Arbor, MI United States; ^10^ Center for Computational Medicine and Bioinformatics University of Michigan Ann Arbor, MI United States; ^11^ Bone Marrow Transplantation Program University of Michigan Ann Arbor, MI United States

**Keywords:** health IT, caregiver, activation, engagement, pediatric, hematopoietic cell transplantation

## Abstract

**Background:**

Pediatric hematopoietic cell transplantation (HCT), commonly referred to as blood and marrow transplantation (BMT), is an intense treatment modality that requires the involvement of engaged caregivers during the patient’s (child’s) prolonged hospitalization. The ubiquity of electronic health records (EHRs) and a trend toward patient-centered care could allow a novel health information technology (IT) system to increase parental engagement. The paucity of research on acute care, hospital-based (inpatient) health IT applications for patients or caregivers provides an opportunity for testing the feasibility of such applications. The pediatric BMT population represents an ideal patient group to conduct an evaluation due to the lengthy inpatient stays and a heightened need for patient activation.

**Objective:**

The primary objective of this study is to assess the feasibility of implementing the BMT Roadmap in caregivers as an intervention during their child’s inpatient hospitalization. The BMT Roadmap is an inpatient portal prototype optimized for tablet with a user-centered design. It integrates patient-specific laboratory and medication data from the EHR in real-time and provides support in terms of discharge goals, home care education, and other components. Feasibility will be proven if (1) the BMT Roadmap functions and can be managed by the study team without unexpected effort, (2) the system is accessed by users at a defined minimum threshold, and (3) the qualitative and quantitative research conducted provides quality data that address the perceived usefulness of the BMT Roadmap and could inform a study in a larger sample size.

**Methods:**

This will be a single-arm, nonrandomized feasibility study. We aim to enroll 10 adult caregivers (age ≥ 18 years) of pediatric patients (aged 0-25 years) undergoing autologous (self-donor) or allogeneic (alternative donor) BMT. Assenting minors (aged 10-18) will also be invited to participate. Recruitment of study participants will take place in the outpatient pediatric BMT clinic. After signing an informed consent, the research study team will provide participants with the BMT Roadmap, available on an Apple iPad, which will used throughout the inpatient hospitalization. To measure the study outcomes, approximately 6-8 semistructured qualitative interviews will be conducted periodically from pre-BMT to 100 days post-BMT and an additional 15-20 semistructured interviews will be conducted among BMT health care providers to assess perceived usefulness and usability of the system, as well as any associated workflow impacts. Quantitative survey instruments will only be administered to adult participants (age ≥ 18 years).

**Results:**

Recruitment will begin in September 2015, and preliminary findings are expected in 2016.

**Conclusions:**

This protocol offers a framework for the design and analysis of a personalized health IT system that has the potential to increase patient and caregiver engagement in acute care, hospital-based contexts.

## Introduction

A growing trend in patient-centered care and health information technology (IT) systems for self-management has led to innovation and better outcomes [1,2]. However, the inpatient setting has seen little advancement in this realm of patient or caregiver engagement. An opportunity exists to capitalize on the ubiquity of electronic health records (EHRs) to provide patients and caregivers real-time access to their own data in the hospital setting [3]. Hematopoietic cell transplantation (HCT), commonly referred to as blood and marrow transplantation (BMT), is an intense treatment modality that requires patients and caregivers to be engaged throughout the prolonged hospitalization, which can last up to 6 weeks. Due to the high-risk and long-term nature, pediatric BMT represents an ideal population for testing the feasibility of novel health IT systems.

### Health Information Technology-Mediated Systems

Health IT systems used during hospitalization, particularly in high-risk diseases such as BMT, could improve clinician-participant engagement [2,4]. Such systems offer the potential to overcome constraints in health care delivery limited by provider time, complicated health information, and financial pressures. Now that the vast majority of acute care hospitals in the United States have an EHR [3], a tailored or personalized health IT system is an accessible option to engage caregivers with their child’s health information [2]. The use of systems that integrate with EHR data would allow for caregiver empowerment in the BMT and other clinical environments. However, there remains a paucity of research on the use of health IT tools in hospital-based contexts. Utilizing qualitative research on the needs of the BMT caregiver population, this study supports a feasibility evaluation of a health IT system, the BMT Roadmap.

### Blood and Marrow Transplantation

BMT is a potentially curative therapy for many malignant and nonmalignant hematologic conditions [5]. Despite advances over the past decade, which have led to improved outcomes, BMT is an intense treatment modality often of last resort for many conditions. A prolonged hospitalization is typically required, keeping patients and their caregivers at the hospital for up to a month or longer [6]. Patients often have a compromised immune system for months following discharge from the hospital, requiring vigilant disease management by families because of its associated high risk for transplant-related mortality [7].

### Patient and Caregiver (Participant) Engagement

Effective communication between clinician-participants is essential in the delivery of health care and is known to impact clinical outcomes [8-10]. Research has shown that participants who are more activated are more likely to engage in disease-specific self-management behaviors and communicate more effectively with providers [11,12]. Moreover, an environment that supports the role of self-concept is more likely to have effective self-managers [13,14].

For critically ill children, such as in the BMT population, caregiver participation is essential [12]. In fact, BMT treatment mandates an identified caregiver [7]. Caregivers, the majority of whom are the mothers of the patients, often take a highly active role in supporting their children throughout the course of hospitalization and beyond [15]. The severity of the illness and high risk of the BMT treatment creates a highly complex self-management environment. While helping their children navigate treatment, caregivers often face major challenges in their social, financial, and emotional lives [16]. This highlights the importance of effective strategies to increase patient-caregiver activation through caregiver empowerment [11]. Empowerment can be increased by reducing the asymmetry of health information between health care providers and caregivers [8,17], which follows a trend toward patient-centered care [18,19].

## Methods

This is a single-arm, nonrandomized study that will take place during the period of hospitalization for either allogenic or autologous BMT. Participants will be provided with the BMT Roadmap system at the time of admission and will be allowed to use it during their length of stay.

### Study Design, Development, and Prototype Testing

**Figure 1:**
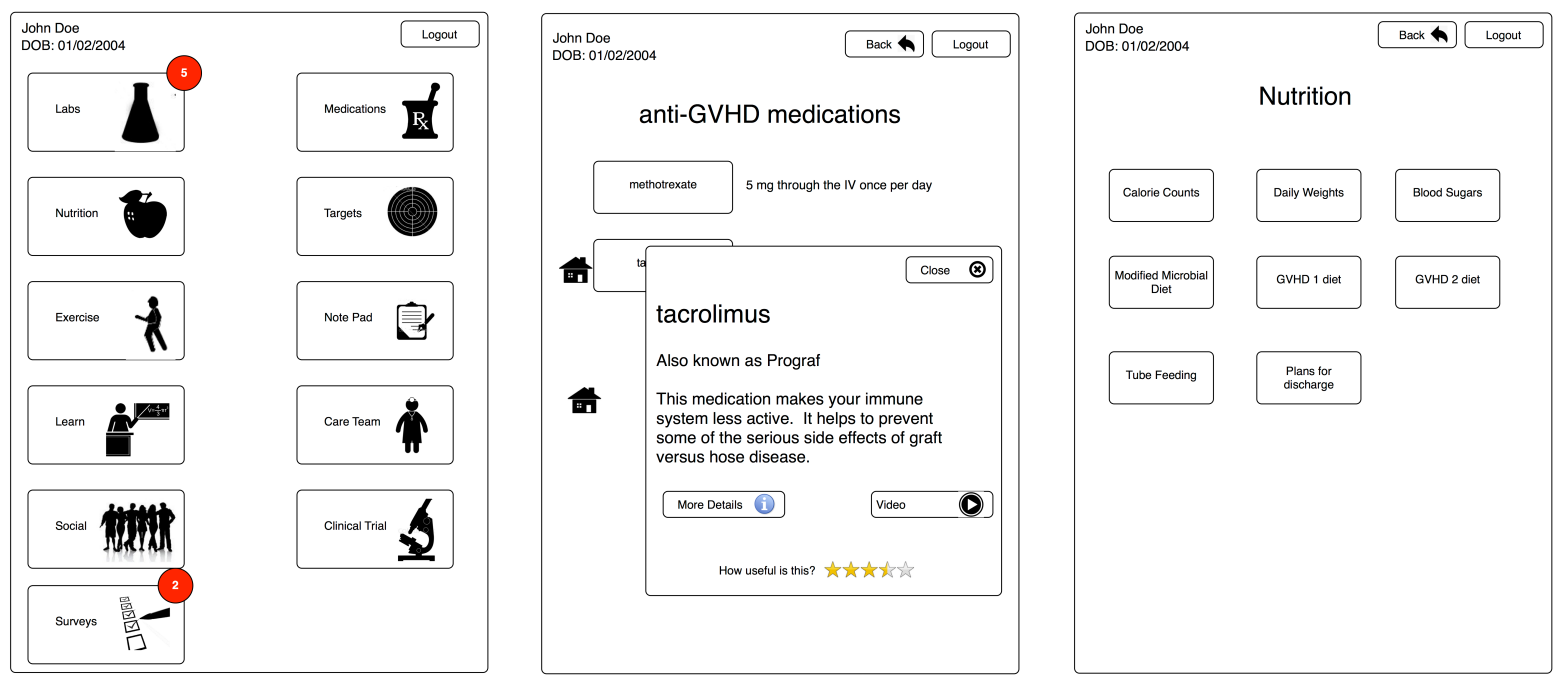
Original concept wireframes.

**Figure 2:**
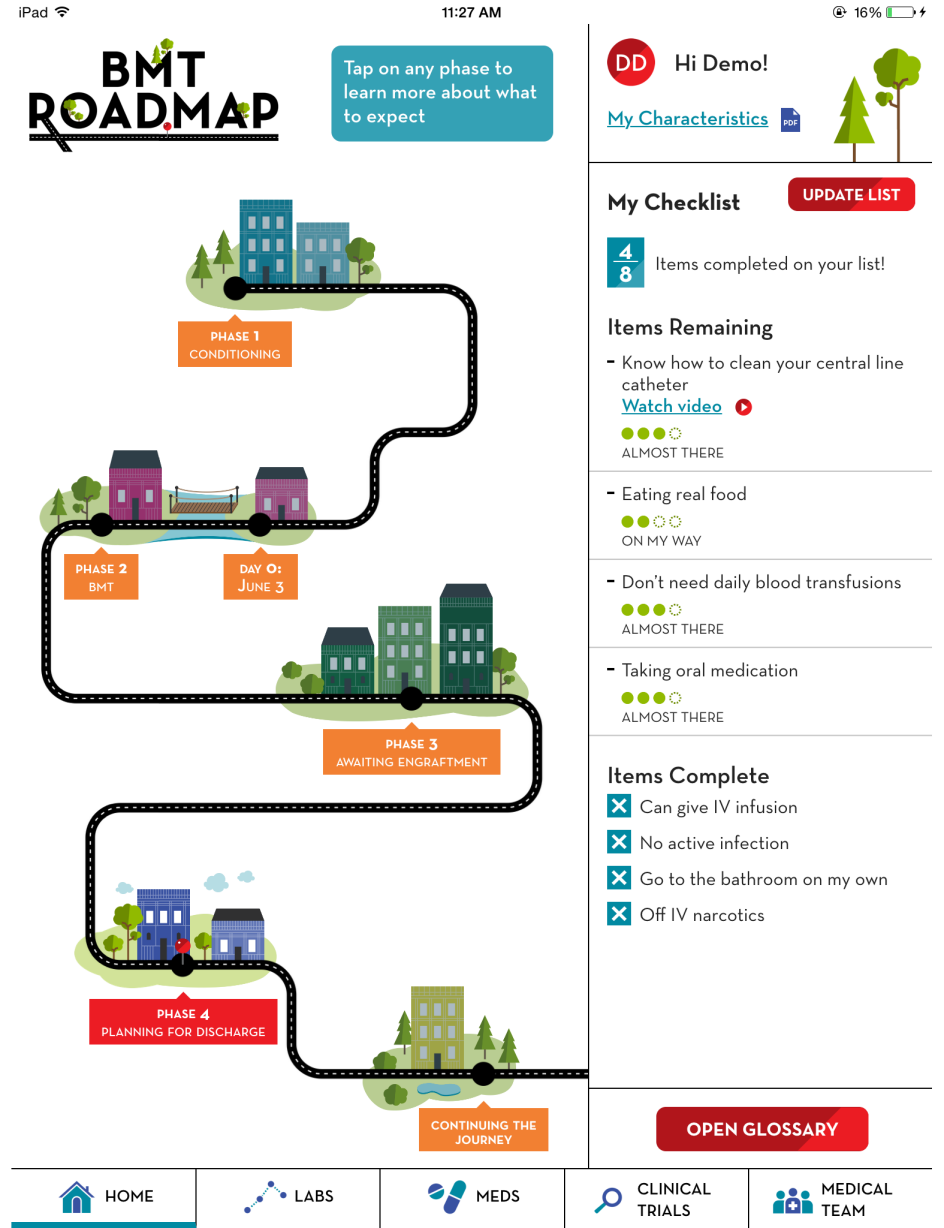
BMT Roadmap home screen, user interface metaphor and discharge criteria.

The design, refinement, and development of the BMT Roadmap were based on user-centered design techniques that incorporated feedback from patients, caregivers, and health care providers [20-23]. Previous ethnographic research incorporated the exploratory evaluation of original concept wireframes to optimize functionality and design (Figure 1) [21,23]. The BMT Roadmap addresses 3 areas in which increased access to information may reduce stress experienced by parent caregivers: navigating the health system and medical communications, managing the day-to-day challenges of routine care, and transitioning to long-term outpatient management. The BMT Roadmap addresses these with 5 content modules personalized to the patients: (1) laboratory studies, (2) inpatient medications, (3) enrolled clinical trials, (4) list of the health care team, and (5) criteria for discharge. The criteria for discharge are presented via both the general progression of inpatient recovery (the “phases” of the BMT Roadmap) and the direct checklist (Table 1). The overall user interface metaphor of a “road map” was selected to represent the experience of BMT patients and caregivers, which encompasses multiple, distinguishable periods. It also references the intended purpose of the tool, to assist caregivers in navigating their child’s BMT journey (Figure 2).

The BMT Roadmap is a Web application that integrates patient-specific data from our vendor EHR, Epic via Web services (Epic Systems Corporation, Verona, WI, USA). Following the wireframes informed through ethnographic research, design and programming work was done by the Center for Health Communications Research (CHCR) at the University of Michigan. During the development, certain functionalities were selected due to prioritized information areas, need for additional research, and feasibility of creation within available resources. Specifically, the BMT Roadmap tool that will be studied under this protocol does not include customizable goals, nutrition, or activities tracking that were explored in early stage, low-fidelity prototypes. The team felt that the selected functionality for this version of the BMT Roadmap represented the most important discovered information areas for the period of hospitalization and allowed for development within a reasonable use of resources. Future research and development will address the additional desires and areas of information management relevant to our patient-caregiver population. The application was optimized to be displayed on and used with an Apple iPad Air. The Apple iPad was chosen based on its familiar gesture-based interface, with which all of the BMT Roadmap operates, and its ergonomics. Additionally, our hospital’s medical IT department could provide support for securely connecting an Apple iPad to our internal hospital wireless network. In addition to the CHCR team, development was collaboratively directed by the research team that encompasses several disciplines including expertise in BMT, computer-supported cooperative work, human-computer interaction, health informatics, survey methodology, and biostatistics.

Two “design workshops” (usability testing) were conducted (unpublished data, March and April 2015) to assess heuristic usability and perceived usefulness of the BMT Roadmap in patients and caregivers. A total of 10 families participated, including 10 caregivers and 7 patients. All families had previously been through the initial hospitalization associated with the BMT procedure and were currently receiving outpatient care. In Workshop 1, participants were given low-fidelity paper prototypes [23]. In Workshop 2, participants evaluated high-fidelity prototypes (wireframes on iPad devices). Participants were directly asked to respond to the user interface metaphor and overall tone of the BMT Roadmap. Overall, responses were positive and the team felt comfortable utilizing the metaphor in the final BMT Roadmap, with the intention to further consider user opinions under this protocol. The 5 modules (eg, labs, medications, clinical trials, health care providers, and discharge criteria) were tested in these families and additional input was sought related to information and caregiving themes. Observation notes and transcriptions from audio recordings were reviewed and discussed by the research study team (MM, DAH, EK, MA, LA, and SWC). Minor changes in the design, language, and functionality were made to reflect expressed concerns in usability. Major changes were documented as potential future functionality or modules not represented in this pilot BMT Roadmap system.

**Table 1:** Summary of the module features and addressed information areas.

BMT Roadmap module	Features of module	Addressed information areas		
		Navigating the health system and medical communications	Managing the day-to-day challenges of routine care	Transitioning to long-term outpatient management
Laboratory studies	Synchronously updated labs include complete blood count for entire hospitalization and a 3-day trend for electrolytes and liver panel, reviewed during rounds	X	X	X
Inpatient medications	Details of medications and their purpose, potential side effects, administration modes, and alternative names	X	X	X
Enrolled clinical trials	Includes plain-language summary and scan of the complete signed consent form, including length of participation and description of relevant tasks or specimens	X		X
List of health care team	Ordered alphabetically or by role, includes photo, name, and role	X	X	
Criteria for discharge (home screen)	Description of the “phases” of BMT that lead toward discharge (what to expect and when)	X	X	X
	Educational videos regarding practices to be done after discharge (safe maintenance of a central line catheter)			X
	Checklist of discharge criteria with bidirectional, 4-point progression slider; intended to set both expectations and task-oriented goals	X	X	X
	Glossary of common terms, available in a list and hyperlinked throughout content	X	X	X
	My Characteristics PDF—a patient-specific summary document frequently referenced by the medical team	X	X	

### Description of Content Features

#### Clinical Phase Descriptions

Starting with chemotherapeutic conditioning before transplant, the BMT Roadmap lays out a progression that patients and caregivers can expect to go through during their HCT experience for families. This is done using graphics of a road map with buildings along the route as a visual metaphor for the major clinical events to be encountered during the treatment course. Each building has a description of potential symptoms or side effects and recovery techniques. By setting expectations in current and future phases, the aim is for families to have more advanced knowledge of the expected course of treatment and the progress one must make before discharge (Figure 2).

#### Patient Characteristics

Each patient will have a personalized “My BMT Characteristics” section that is developed by the health care team. This form will provide information unique to the patient undergoing BMT, such as type of conditioning chemotherapy regimen, dates of the procedure, infectious disease markers, blood type, etc.

#### Discharge Criteria

Discharge progress is represented alongside phases in the BMT Roadmap, describing general health and emotional and progress expectations. Discharge criteria are presented in the form of a to-do list. The caregivers will have the ability to note their progress for each element to mark when they are closer to completing the criteria using a bidirectional, 4-point sliding scale to reflect the fluidity of medical progress. Items also include educational tools, including self-care instructional videos for posthospitalization (Figure 2).

#### Medication List

All prescribed inpatient medications are displayed, grouped by purpose (eg, pain control, antibiotic, antigraft-vs-host disease), including the dose, method of administration, and side effects.

#### Lab Results

Lab results are updated from the EHR in real-time and offer basic visualizations of trends, as well as the most recent result in numeric form. The primary results of importance to these patients are provided including blood counts, and electrolyte and liver panels.

#### Medical Team

Because families have difficulty identifying and remembering the numerous people providing inpatient care throughout the day, we provide names and photographs of the care team, including roles (eg, nurse practitioner, attending physician, fellow).

#### Clinical Trials

Families also often forget what clinical trials they are enrolled in. Therefore, individualized clinical trial enrollment information can be provided for each patient, including a descriptive overview of the study, study details, and a scan of the consent form that was signed.

#### Glossary

The glossary includes commonly used terms and their definition. The glossary can be accessed on the home screen or by clicking on hyperlinked terms throughout the application.

### Security Measures

Health information data displayed by the system will include patient laboratory results, medications, and a copy of signed informed consent documents. While this protected health information will be displayed on the iPad, the data are not stored anywhere within the system. Rather, the latest results will be retrieved in real time using Web services to the EHR and displayed to the user. Users are authenticated using a username and password. Passwords are stored in the system using a secure, 1-way salted hashing function. Access to the underlying database is restricted to the host server only. Detailed audit trails of use are maintained. Servers will be hosted by the health system’s medical center information technology (MCIT) department behind the institution’s firewall, within the highly secure medical center data center. The iPads themselves are specially configured by MCIT to automatically connect to the hospital’s secure, encrypted wireless network and to limit access to certain applications and URLs within the Web browser; users will not be able to install their own apps. This device will not work if removed from the hospital wireless network and can be deactivated remotely. Importantly, the security provisions for this application were thoroughly reviewed and vetted by the medical school’s information services department using a formal risk assessment template approved by the health system’s compliance office.

Patient and caregiver accounts will be created by the study team users through a secure management console accessible only by staff users. Patient and caregiver accounts are generated for users who have consented to be a part of the study, allowing for user-created password and a security question. The application times out after 10 minutes, requiring re-entry of the password. After 4 unsuccessful password attempts, account reactivation requires an administrator override. All functions require an authenticated user. Protected health information will be identified with access only provided to authorized personnel. The BMT Roadmap information system does not have any trust relationships with external environments (eg, interconnection agreements with third parties).

### Participant Eligibility, Recruitment, and Accrual

Participants will be limited to those hospitalized for a first experience with BMT. Eligibility includes cancer and noncancer, autologous or allogeneic transplant cases. Any consenting adult caregiver (age ≥18 years) or minor assenting patient (aged 10-18 years with caregiver consent) is able to participate in the study.

Based on the annual BMT census at the University of Michigan, 10 caregiver participants or patient-caregiver pairs (in the case of an assenting minor) will be recruited at the rate of 2-3 participants or pairs a month. All candidates for BMT are discussed at a weekly new patient evaluation meeting, which will help the study team identify potential study participants.

Health care providers who work in the pediatric BMT unit will also participate in research observation and interviews related to their experience as patients or caregivers use the BMT Roadmap.

### Objectives

We hypothesize that the BMT Roadmap could provide a platform to promote caregiver (parent) activation and enhance health communication in a hospital-based context. Feasibility of the BMT Roadmap will be evaluated by considering outcomes of the following objectives:

1. Test the rate BMT Roadmap use in this population, with an expected threshold of 20% caregiver or patient enrollment into the study, with use throughout their hospitalization, based on at least a single login per day threshold.
2. Evaluate the impact of the BMT Roadmap system on caregiver activation, satisfaction, and burden through use of a survey instrument (eg, parent-patient activation model). To account for potential confounding, anxiety, mood, stress, miscarried helping, and experiential avoidance will also be measured through validated survey measurements instruments (Table 2).
3. Evaluate the usefulness and usability of the BMT Roadmap (in participants and health care providers) through a validated survey instrument (Table 2).
4. Assess the attitudes and perceptions of the participants and health care professionals on the BMT Roadmap through qualitative interviews.
5. Identify the presence and quality of care process redesign associated with the BMT Roadmap.

**Table 2:** Survey instrument summary.

Measures	Instrument	Description	Population	Time (min)	Baseline	Discharges	Day 100
Activation	Parent-PAM [12]	Modified version of PAM, which was developed to study parental activation using physical, emotional, and role domains of general functioning	Caregivers	5-7	X	X	X
Satisfaction	Press Ganey [24]	Patient inpatient experience and overall satisfaction	Caregivers	5-7		X	X
Anxiety	State Trait Anxiety Inventory [25]	Commonly used and validated tool with good test-retest reliability	Caregivers	5	X	X	X
Mood	Profile of Mood States [26]	Assess transient distinct mood states with 6 factor-based subscales: tension/anxiety, depression/dejection, anger/hostility, fatigue/inertia, vigor/activity, and confusion/bewilderment	Caregivers	5-7	X	X	X
Burden	Caregiver Quality of Life—Cancer [27]	35-item rating scale measuring physical, emotional, family, and social functioning burden will also be used to evaluate caregiver burden	Caregivers	8-10	X	X	X
Stress	Impact of Event-Revised [28,29]	22-item scale measuring difficulty faced over the past 7 days; the score may be reflective of an acute stress disorder	Caregivers	5-7	X	X	X
Miscarried helping	Helping for Health Inventory [30]	15-item scale to assess how caregivers perceive their own communication style regarding the patient’s chronic illness; assesses miscarried helping (ie, how the well-intentioned efforts of caregivers of children with chronic diseases may become barriers to successful treatment)	Caregivers	5	X	X	X
Experiential avoidance	Parental Acceptance and Action Questionnaire (PAAQ) [31]	15-item scale that assesses experiential avoidance within the caregiver role. The PAAQ includes 2 factors: "inaction," composed of 9-items; and "unwillingness," composed of the remaining 6-items	Caregivers	5	X	X	X
Usefulness	Usefulness [32]	Custom questionnaire, as there are no validated instruments for measuring satisfaction specific to the BMT Roadmap information system	Caregivers, health care professionals	2	X	X	X
Usability	Usability [32]	Custom questionnaire, as there are no validated instruments for measuring satisfaction specific to the BMT Roadmap information system	Caregivers, Health care professionals	2	X	X	X

### Outcomes Measures

Qualitative and quantitative research methods will be used to evaluate the objectives. From the time of admission until 100-days post-transplant, which is considered an end point for early recovery from HCT, the research team will conduct semistructured, qualitative interviews and administer validated surveys (Table 2). All interviews will be audio recorded.

Approximately 6-8 semistructured interviews per participant will probe around broad caregiver information needs, the perceived usefulness of the tool, and any literacy or usability feedback regarding the tool or its presentation on an Apple iPad. Participants will be encouraged to “think aloud” and will be prompted to complete particular tasks or offer understanding of specific information based upon previous interviews. Survey instruments allow for evaluation of outcome measures, such as usability of the tool and caregiver activation level, as well as potentially confounding factors, such as stress or mood (Table 2).

Another 15-20 qualitative interviews will be conducted of BMT health care providers, including physicians, nurses, pharmacists, nutritionists, social workers, and psychologists. Health care professionals will complete a subset of the surveys. Both interviews and surveys will seek opinions on the usefulness or usability of the tool from a provider’s perspective, as well as the presence and quality of care process redesign in response to patients’ or caregivers’ use of the tool.

Additional data collected include demographic and baseline information, comprehensive system usage logs (eg, login frequency, pages viewed on the BMT Roadmap information system, time stamps) and clinical outcomes (eg, length of stay, risk of day 30 and 100 readmission, infections, transplant-related mortality, and survival).

### Analyses

Qualitative interviews will undergo systematic, transcript-based analyses. Observational field notes will complement the recorded audio. Each session will be transcribed and coded to identify trends, adding additional codes and adjusting interview structure based on initial results.

Analysis of the survey instrument data will include a reference population of Parental-PAM (P-PAM) scores available in the pediatric BMT caregiver population that measures activation/participation. Comparisons of proportions of the study population to the reference population will be taken into account. Descriptive statistics will be calculated for each P-PAM score and stratified into the appropriate level of activation. Results will be compared with a published sample [12]. Univariate analyses will be performed to assess associations between P-PAM and demographic, social, and environmental characteristics of the parent (eg, type of insurance, marital status, number of children in household), disease-related characteristics of the patients (eg, age, disease, disease status at BMT), satisfaction [24], usefulness and usability [32], caregiver burden [27], mood [26], anxiety [25], stress [28], miscarried helping [30], and experiential avoidance [31]. Pearson’s correlation (and other suitable measures of association for categorical variables) will be used to determine the nature and significance of association between each variable and Parent-PAM scores. Despite limited degrees of freedom due to the sample size, the survey instrument data will be analyzed using methods appropriate to the scale of items.

An exploratory analysis of system usage logs and clinical outcomes will be conducted to find potential correlations worthy of further study or that would inform future design of a personalized information system.

### Pilot Study, Sample Size, and Retention

A sample size of 10 adult caregivers allows for an exploratory and feasibility study on the use of the BMT Roadmap at our institution. Recognizing that people have varying levels of technology skills, retention will be determined by demonstrating maintenance of at least 60% of patients and caregivers who agree to using the BMT Roadmap throughout inpatient hospitalization (ie, they log in at least once daily). By conducting interviews and surveys during routine hospital and outpatient care, there are minimal anticipated missing data. Given the sample size and the single-arm nature of the study, we will not be powered to determine statistically significant results in many outcome measures. However, the data within an approximate 1-year study period are expected to inform a future clinical trial and improved design of the BMT Roadmap.

## Results

Recruitment will begin in July 2015 and results are expected in 2016. The evidence will reveal the feasibility of utilizing an educational health IT tool in the BMT hospitalization process.

## Discussion

The goal of this pilot study is to evaluate the feasibility of implementing a patient-centric, personalized health IT system in the hospital-based BMT setting. In this case, feasibility will be proven if (1) the BMT Roadmap functions and can be managed by the study team without unexpected effort, (2) the system is accessed by users at a defined minimum threshold, and (3) the qualitative and quantitative research conducted provides quality data that addresses the perceived usefulness of the BMT Roadmap and could inform a study in a larger sample size. We hypothesize that caregivers will choose to use the BMT Roadmap, report overall satisfaction with the system, and become active participants in their health care. It is possible that health care providers in the hospital unit will be satisfied with the BMT Roadmap and its impact on the clinician-participant relationship.

### Conclusions

This protocol offers a framework for design and analysis of personalized health IT systems to increase patient and caregiver engagement in acute care, hospital-based contexts.

## Abbreviations

BMT: blood and marrow transplantation
CHCR: Center for Health Communications Research
HCT: hematopoietic cell transplantation
EHR: electronic health record
IT: information technology
MCIT: medical center information technology
PAAQ: Parental Acceptance and Action Questionnaire
P-PAM: Parental-PAM

## References

[1] Kimball A, Corey K, Kvedar J (2015). ; Accessed February 24.

[2] Prey J, Woollen J, Wilcox L, Sackeim A D, Hripcsak G, Bakken S, Restaino S, Feiner S, Vawdrey D (2014). Patient engagement in the inpatient setting: a systematic review. J Am Med Inform Assoc.

[3] Charles D, Gabriel M, Furukawa M (2014). ONC Data Brief; No 16; May.

[4] Ricciardi L, Mostashari F, Murphy J, Daniel JG, Siminerio EP (2013). A national action plan to support consumer engagement via e-health. Health Aff (Millwood).

[5] Copelan EA (2006). Hematopoietic stem-cell transplantation. N Engl J Med.

[6] Ford R, Wickline M, Appelbaum FR, Forman SJ, Negrin RS, Blume KG (2009). Nursing Role in Hematopoietic Cell Transplantation. Thomas' Hematopoietic Cell Transplantation.

[7] Gemmill Robin, Cooke Liz, Williams Anna Cathy, Grant Marcia (2011). Informal caregivers of hematopoietic cell transplant patients: a review and recommendations for interventions and research. Cancer Nurs.

[8] Cegala DJ, Post DM (2009). The impact of patients' participation on physicians' patient-centered communication. Patient Educ Couns.

[9] Institute OM (2001). Crossing the quality chasm: A new health system for the 21st century.

[10] Quinn GP, Gwede CK, Cases MG, Barata A, Cessna J, Christie J, Gonzalez L, Koskan A, Pidala J, Jim H S L (2014). Patient education in allogeneic hematopoietic cell transplant: what patients wish they had known about quality of life. Bone Marrow Transplant.

[11] Hibbard Judith H, Mahoney Eldon R, Stock Ronald, Tusler Martin (2007). Do increases in patient activation result in improved self-management behaviors?. Health Serv Res.

[12] Pennarola Brian W, Rodday Angie Mae, Mayer Deborah K, Ratichek Sara J, Davies Stella M, Syrjala Karen L, Patel Sunita, Bingen Kristin, Kupst Mary Jo, Schwartz Lisa, Guinan Eva C, Hibbard Judith H, Parsons Susan K, Hsct- CHESSStudy (2012). Factors associated with parental activation in pediatric hematopoietic stem cell transplant. Med Care Res Rev.

[13] Becker ER, Roblin DW (2008). Translating primary care practice climate into patient activation: the role of patient trust in physician. Med Care.

[14] Fowles JB, Terry P, Xi M, Hibbard J, Bloom CT, Harvey L (2009). Measuring self-management of patients' and employees' health: further validation of the Patient Activation Measure (PAM) based on its relation to employee characteristics. Patient Educ Couns.

[15] Rehm RS (2013). Nursing's contribution to research about parenting children with complex chronic conditions: an integrative review, 2002 to 2012. Nurs Outlook.

[16] Phipps S, Dunavant M, Lensing S, Rai SN (2004). Patterns of distress in parents of children undergoing stem cell transplantation. Pediatr Blood Cancer.

[17] Gentles Stephen James, Lokker Cynthia, McKibbon K Ann (2010). Health information technology to facilitate communication involving health care providers, caregivers, and pediatric patients: a scoping review. J Med Internet Res.

[18] Epstein R, Street R (2007). Patient-Centered Communication in Cancer Care: Promoting Healing and Reducing Suffering. NIH Publication No. 07-6225.

[19] Mead N, Bower P (2000). Patient-centredness: a conceptual framework and review of the empirical literature. Soc Sci Med.

[20] Keusch F, Rao R, Chang L, Lepkowski J, Reddy P, Choi SW (2014). Participation in clinical research: perspectives of adult patients and parents of pediatric patients undergoing hematopoietic stem cell transplantation. Biol Blood Marrow Transplant.

[21] Kaziunas E, Buyuktur AG, Jones J, Choi SW, Hanauer DA, Ackerman MS (2015). Transition and Reflection in the Use of Health Information: The Case of Pediatric Bone Marrow Transplant Caregivers.

[22] Büyüktür A, Ackerman M (2014). Issues and opportunities in transitions from speciality care: a field study of bone marrow transplant. Behaviour & Information Technology.

[23] Kaziunas E, Hanauer D, Ackerman M, Choi S (2015). Identifying unmet information needs in the inpatient setting to increase patient and caregiver engagement in the context of pediatric hematopoietic stem cell transplantation. Journal of American Medical Informatics Association. In press. PMID.

[24] Jha AK, Orav EJ, Zheng J, Epstein AM (2008). Patients' perception of hospital care in the United States. N Engl J Med.

[25] Spielberger C, Gorsuch R, Lushene R, Vagg P, Jacons G (1983). Manual for the State-Trait Anxiety Inventory.

[26] McNair D, Loor M, Droppelman L (1981). Profile of Mood States.

[27] Weitzner MA, Jacobsen PB, Wagner H, Friedland J, Cox C (1999). The Caregiver Quality of Life Index-Cancer (CQOLC) scale: development and validation of an instrument to measure quality of life of the family caregiver of patients with cancer. Qual Life Res.

[28] Weiss D, Marmar C (1997). Impact of Event Scale-Revised. In P Wilson & T Keane (Eds), Assessing psychological trauma and post traumatic stress disorder: A handbook for practitioners. Vol.

[29] Perrin S, Meiser-Stedman R, Smith P (2005). The Children's Revised Impact of Event Scale (CRIES): Validity as a Screening Instrument for PTSD. Behav. Cognit. Psychother.

[30] Harris M, Antal H, Oelbaum R, Buckloh L, White N, Wysocki T (2008). Good intentions gone awry: Assessing parental "miscarried helping" in diabetes. Families, Systems, & Health.

[31] Cheron DM, Ehrenreich JT, Pincus DB (2009). Assessment of parental experiential avoidance in a clinical sample of children with anxiety disorders. Child Psychiatry Hum Dev.

[32] Davis F (1989). Perceived Usefulness, Perceived Ease of Use, and User Acceptance of Information Technology. MIS Quarterly.

